# Measuring the effectiveness of digital nursing technologies: development of a comprehensive digital nursing technology outcome framework based on a scoping review

**DOI:** 10.1186/s12913-020-05106-8

**Published:** 2020-03-24

**Authors:** Tobias Krick, Kai Huter, Kathrin Seibert, Dominik Domhoff, Karin Wolf-Ostermann

**Affiliations:** 1 SOCIUM Research Center on Inequality and Social Policy, University of Bremen, Mary-Somerville-Straße 3, 28359 Bremen, Germany; 2 High-profile Area of Health Sciences, University of Bremen, 28359 Bremen, Germany; 3 Institute for Public Health and Nursing Research, University of Bremen, Grazer Straße 4, 28359 Bremen, Germany

**Keywords:** Technology, Care, Nursing, Framework, Effectiveness, Effect, Digital, Outcome, Evidence

## Abstract

**Background:**

Digital nursing technologies (DNT) comprise an expanding, highly diverse field of research, explored using a wide variety of methods and tools. Study results are therefore difficult to compare, which raises the question how effectiveness of DNT can be adequately measured. Methods currently used might not be sufficient for certain specific nursing contexts. A comprehensive outcome framework that shows the multitude of possible outcome areas could be useful to generate more comparable results. The aim of the present study is to develop an outcome framework for DNT and to indicate which outcome areas have been most frequently evaluated in previous studies and how this has been done.

**Methods:**

We combined an inductive and deductive approach to develop the framework. The numerical analysis is based on a scoping review focussing on the effectiveness of DNT for persons in need of care, formal or informal caregivers or care institutions. Nine databases were included in the screening: Medline, Scopus, CINAHL, Cochrane Library, ACM Digital Library, IEEE Xplore, the Collection of Computer Science Bibliographies, GeroLit and CareLit. Additional literature searches and expert interviews were included.

**Results:**

The developed framework comprises four outcome target groups and 47 outcome areas. There are considerable differences in the researched outcome areas for the individual outcome target groups. Persons in need of care were by far the most frequently surveyed, particularly with respect to their psychological health. There are much fewer studies on formal and informal caregivers, and it is particularly noticeable that the quality of life of both groups has rarely been investigated. Care process quality was most frequently researched for organisations.

**Conclusion:**

We were able to provide a comprehensive DNT outcome framework, thereby identifying the outcome tools used and the less researched outcome areas. We recommend a detailed investigation of all areas and tools in future research projects with a view to initiating a discussion on the differing importance of existing outcome areas and on a standardisation of outcome tools. We also recommend the development of outcome areas for the macro level of effectiveness assessment.

## Background

Research on digital nursing technologies is an emerging field. An initial analysis of 715 articles on Digital nursing technologies (DNT) showed the existing variety of research. The field is highly diverse and is explored using a multitude of methods and instruments, which makes the measured effects very difficult to compare [1]. The question how effectiveness of DNT can be adequately measured is becoming increasingly relevant. There are no comprehensive systematisations that can help to structure this measurement, which is why only frameworks that cover partial areas [2] of the field or from other related healthcare contexts such as eHealth [3] or HTA [4] can be used.

Many systematic reviews in the field of technology and nursing conclude that solid evidence with respect to effectiveness is lacking [5–12], mainly due to a weak level of evidence and the incomparability of the study results. For this reason, a comprehensive outcome framework detailing the multitude of possible outcome areas could be useful for generating more comparable results. A systematisation of outcome areas and outcome tools for DNT could promote scientific exchange, improve the comparability of results and facilitate the identification of research gaps. Hence, the aim of this research is to systematically develop a comprehensive outcome framework for DNT and to indicate which outcome areas have been most frequently evaluated in previous studies, and on this basis to provide an overview on research focuses and possible gaps in current research on DNT. The development of the framework and the mapping of current research are based on a scoping review. The categories of the framework were further elaborated on the basis of additional literature and expert knowledge.

Other frameworks, like the evaluation framework for Health Information Systems (HOT-fit) [13], the General Framework for Evaluating Health Information Technology [14], the Canadian Health Information Performance Framework [15], the OECD Framework for Health System Performance Measurement [16], the Infoway Benefits Framework [17] or the MAST Framework [18], have been proposed to structure the process of effectiveness evaluation in health care areas using digital technologies. However, the existing frameworks and categorization systems have different focal points, and none of them are – from our point of view – adequately geared to the specific needs of the complex nursing care context. Outcomes relating to caregivers, and in particular informal caregivers, are often neglected or overlooked. The Infoway Benefits Evaluation Framework [17] that has been proposed for the analysis of health information systems in Canada, and the Model for Assessment of Telemedicine (MAST) [18], pose an exception here, as they are very well elaborated. Both frameworks were developed in a similar context, but for different purposes. The frameworks do not specifically consider informal caregivers, but they nevertheless indicate important sub-areas for the evaluation of DNT (such as, for example, patient safety, care quality, access to care or organisational productivity) that should be integrated into a comprehensive DNT framework. However, they are inadequate and inappropriate for a specific application relating to DNT, because they have been developed for different purposes. In addition to these two frameworks, there is also a framework which especially displays the impact of ICT solutions on nursing care. The adapted version of the Nursing Care Performance Framework [2] focusses on organisational issues, formal caregivers and aspects relating to people in need of care. This makes a helpful contribution in these areas. However, outcomes for technologies on informal caregivers are not represented in this framework. Besides these frameworks, there is also specific work on the measurement of the effectiveness of technology for ageing people in general, including effects on physical and psychological health, mobility, social connectedness, safety, everyday activities and leisure [19].

As we have established, therefore, a number of different instruments for categorizing outcome measures and tools in healthcare contexts have already been developed. To the best of our knowledge, however, there is no extensive analysis of relevant outcome areas and outcome tools pertaining to digital nursing technology published in the English nursing literature. We therefore decided to develop a new comprehensive outcome framework that is applicable to the design of effectiveness evaluation studies in the field of digital nursing technologies.

### Definitions

In order to elucidate the conceptual differences between the individual terms in this study, we shall first define our understanding of the most important terms. The main subject of this article is digital nursing technologies (DNT). DNT are required i) to support the immediate action of a caregiver (e.g. decision support systems for guideline compliance [20]); ii) to contribute to the self-reliance of the person in need of care in such a way that direct on-site care assistance can be avoided (e.g. ambient assisted living support at home [21]); iii) to substitute the nursing support by using technology (e.g. robot that measures vital signs prior to consultation) [22]; or iv) to support the training or education of nurses (e.g. high fidelity simulator systems [23]) [1]. Technological support may relate to the person in need of care, formal or informal caregivers, or to an organisational process.

We also distinguish between the terms “outcome measure” and “outcome tool”. An outcome measure is a specific measure used to quantify (quantitative) or gauge (qualitative) an effect, e.g., of an eHealth intervention, and an outcome tool is a specific instrument used to collect quantitative or qualitative data [24, 25]. Outcome tools or outcome measures are indicators that represent effects in a specific outcome area. A distinction is also made between the terms “outcome target group” and “outcome area”. The outcome target group refers to the assignment of the outcome of a technology to a specific group of people (e.g. formal caregivers) or to an organisation (e.g. hospital). The outcome area specifies the content layer on which an effect occurs (e.g. well-being or functional health). There is also a distinction between the terms ‘effectiveness’ and ‘efficacy’. Efficacy studies measure (expected) effects under ideal circumstances, effectiveness studies measure (beneficial) effects under “real world” conditions [26]. Since we have found an incoherent use of the terms in the studies included, we use the term “effectiveness” to cover both concepts. This decision will be further justified and discussed in the discussion section.

### Objective and research question

The ultimate objective of this article is to develop an outcome framework for DNT that enables systematic classification into different outcome areas and can be used to support future effectiveness research. It is essential to the development of such a framework that past attempts to evaluate effectiveness in previous studies are understood. This review is thus guided by the following main research questions: (i) Which possible outcome areas for measuring effectiveness of digital nursing technologies can be identified? (ii) Which outcome areas have so far been the focal point of research on effectiveness of digital nursing technologies, and which areas have been researched less frequently or not at all? (iii) How has effectiveness been measured in previous studies?

## Methods

Our analysis is based on a previous scoping review (phase one) [1], which we conducted on the basis of Arksey and O’Malley’s scoping review framework [27]. We used processual advice drawn up by Levac, Colquhoun et al. [28] to enhance the scientific process. This was particularly important because the search of the previous scoping review generated a large number of titles, which made the identification, selection and charting of the relevant studies very time consuming and resource intensive. We have tried to counteract this by using the advice to refine the search and selection strategy in an iterative process. We also jointly developed, tested and updated a data charting form that allowed us to review and extract each full text by one researcher, a second author was consulted in case of uncertainties regarding the classification.

The scoping review included 715 studies focussing on acceptance, effectiveness or efficiency DNT. The full search strategy, analysis and results of the scoping review are published in Krick et al. 2019 [1]. The initial analysis of the scoping review yielded strong indications that a more in-depth analysis of tools and research areas could be useful for further research. Therefore, we decided to extend the evaluation to include methodological questions in a second research phase. In the following, the method of the initial scoping review will be briefly presented, followed by an explanation of the method used in the second phase.

### Methodical foundation of the initial scoping review

We screened 19.510 titles, based on a search in nine electronic databases, covering studies published between 2011 and March 2018. The initial scoping review (phase one) was conducted with a view to a broader research question insofar as studies were included that related to the acceptance, the effectiveness or the efficiency of a digital nursing technology.

### Eligibility criteria of the initial scoping review and the analysis of effectiveness

In order to understand how the preselection of articles for this review took place, the eligibility criteria of Phase one will now be explained briefly. Articles were included if they reported on study results relating to acceptance, effectiveness (on any evidence level) or efficiency (including cost analysis); target settings include residential long-term care, formal and informal care at home, hospital care, palliative inpatient care, intensive care unit care, day-care centre care and cross-sectoral care. Based on this preselection, all studies that reported on the effectiveness of DNT were included for the second phase of analysis. In the next step, as we aimed to focus on effects on persons in need of care, caregivers and care organisations, all studies that aimed primarily at an educational environment and studies conducted in a laboratory environment were excluded. Studies in a laboratory environment were excluded as most of them measure technical effectiveness, which is not the subject of our analysis. The remaining articles were analysed according to the outcome areas, outcome measures and outcome tools to create an empirical basis for the DNT outcome framework. Again, studies were excluded that focussed only on technical effectiveness. Based on these limitations, 123 individual studies were subjected to the analysis presented in this article.

### Development of the outcome framework and data assignment

A combined inductive/deductive approach was used to develop the outcome framework. A basic model was developed by drawing on the analysis of the effectiveness articles (*n* = 123) of the initial scoping review [1]. Two authors screened the full texts to identify all relevant information. The identified outcome measures and outcome tools of the studies were used to inductively derive a preliminary systematic draft of outcome areas. In addition, a narrative literature search was carried out in the databases PubMed and Google Scholar in order to review whether further outcome areas could be found or identified that were not considered in the 123 studies. We combined the search terms “nursing”, “framework”, “outcome”, “digital” and “technology” and decided which articles should be included in the analysis. The search focussed on studies that explicitly referred to specific frameworks, developed frameworks themselves or provided a systematic structure to measure effectiveness. We analysed the texts and reference lists for relevant frameworks or systematisations. This snowballing method is important for such complex search fields. It helped us to provide meaningful additional information as a supplement to the systematic approach [29]. The aim was to identify studies or other frameworks that describe or contain further outcome areas for DNT. All relevant information that could be used to determine further outcome areas was extracted.

Deductive reasoning was used to gather information from general frameworks and inductive reasoning was used for single studies. A definition of each potential outcome area was then drawn up by one author and discussed with the other authors as to their relevance and fit for the framework. The development process included a multi-stage discussion and iteration with multiple revisions to ensure high quality decisions on the outcome areas and their definitions. The outcome framework was then validated by a group of ten German experts in the measurement of effectiveness of DNT. At the time of the survey, the experts in question were all involved in projects evaluating different digital technologies in nursing in the German healthcare system. The experts were requested to report their own project experiences in order to supplement missing relevant outcome areas. This survey took place as part of a regular exchange among experts.

The final outcome areas of the framework described for persons in need of care were developed with strong references to the Nursing Outcomes Classification (NOC) criteria [30] and existing frameworks [17, 18]. The caregiver-related outcome areas were derived from the initial scoping review and supplemented by categories based on the literature review (e.g. [18, 31–38]), while the organisation-related outcome areas emanate from the literature review (e.g. [17, 18, 39–45]), supplemented by expert opinions. All information is included in the final definitions for each outcome area documented in the Additional files 1, 2, 3, 4.

### Data assignment

In the last step of the second phase, the extracted outcome tools from all the studies included were assigned to the outcome areas in the outcome framework using the collectively developed criteria. One researcher re-reviewed all studies (*n* = 123) to ensure a consistent assignment of outcome measures to outcome areas based on the iteratively developed framework. As a result, the numerical analysis can be presented according to the DNT outcome framework.

## Results

### Analysis results

A total of 123 studies from the initial scoping review were included in the analysis. These studies refer to the following technology categories: ICT, robotics, monitoring, sensors, assistive devices, ambient assisted living and virtual reality as defined by Krick et al. (2019) [1]. A more detailed systematization of the included technologies together with a list of all included studies is provided in Additional file 5.

**Fig. 1:**
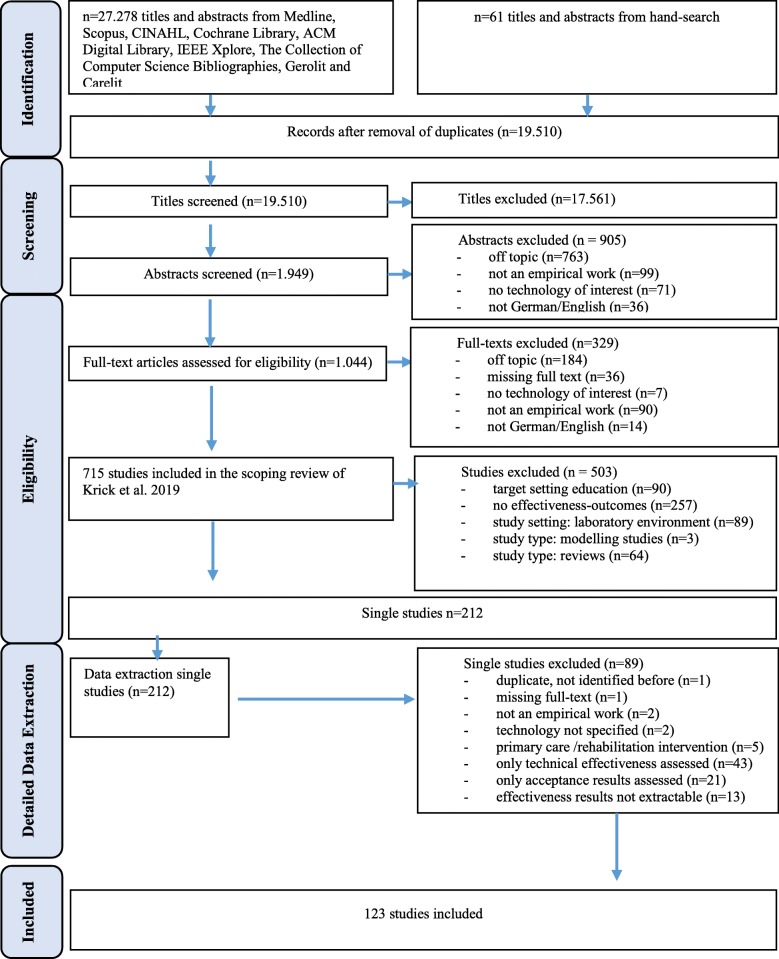
Search results and publication selection process

The PRISMA Flow Chart is presented in Fig. 1.

### Digital nursing technology (DNT) outcome framework

**Fig. 2:**
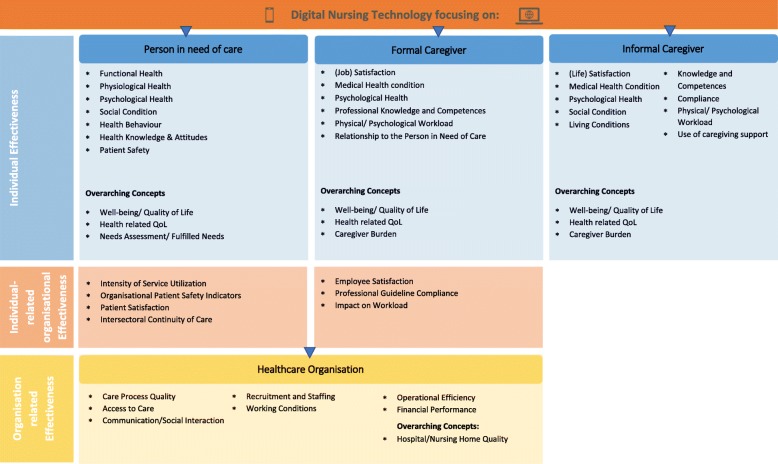
Outcome Framework

We developed an outcome framework to provide a systematic classification and orientation scheme for outcome measures and outcome tools in the field of DNT (Fig. 2). The classification of the framework differentiates between the four outcome target groups: persons in need of care, formal caregivers, informal caregivers and healthcare organisations. For persons in need of care and formal caregivers we also distinguish between individual effectiveness and individual-related organisational effectiveness. This distinction is used to categorize outcome measures or tools that clearly apply to an individual but are closely related to organisational effectiveness and thus in an intermediate area. Overall, the model comprises 47 different outcome areas (e.g. functional health, well-being, patient satisfaction). The outcome areas included in the framework refer to micro (individual) or meso (organisational) levels of evaluation [46]. The macro level is deliberately not included here because it implies different study perspectives and so far, has only been very rarely analysed in the field of DNT. Detailed definitions and examples for the individual categories are provided in Additional files 1, 2, 3, 4.

### Overall outcome areas

This chapter comprises a detailed analysis of all outcome tools and measures used in the 123 studies included (Table 1). The numerical analysis shows which outcome areas have or have not been extensively analysed with the respective measures or tools. Each study that used an outcome tool or outcome measure in a specific area is only included once in this analysis. Most of the included studies investigated the effectiveness of the technologies in question on persons in need of care (*n* = 77). Aspects of organisational effectiveness were measured in 45 studies and only 30 studies referred to caregiver outcomes (formal *n* = 20; informal caregivers *n* = 10). There are substantial differences in the researched outcome areas for the individual outcome target groups. Psychosocial health (*n* = 33), intensity of service utilization (*n* = 19) and organisational patient safety indicators (*n* = 19) for persons in need of care were measured much more frequently than needs assessment/ fulfilled needs (*n* = 3), health knowledge & attitudes (*n* = 3) or intersectoral continuity of care (*n* = 1). For formal caregivers the outcome areas most frequently covered are relationship to person in need of care (*n* = 7), guideline compliance (*n* = 7) and physical/psychological workload (*n* = 6). Well-being/quality of life, health-related quality of life (QOL), medical condition and caregiver burden were not measured once in our sample. The other outcome areas were only rarely analysed.

**Table 1:** Framework with overall results

Person in need of care	N	Formal Caregiver	N	Informal Caregiver	N	Healthcare Organisation	N
Functional Health	16	(Job) Satisfaction	3	(Life) Satisfaction	2	Care Process Quality	21
Physiological Health	11	Medical Health condition	0	Medical Health Condition	0	Access to Care	1
Psychosocial health	33	Psychosocial health	0	Psychosocial Health	4	Communication/Social Interaction	15
Social Condition	7	Professional Knowledge and Competences	2	Social Condition	3	Recruitment and Staffing	0
Health Behaviour	5	Physical/ Psychological Workload	6	Living Conditions	2	Working Conditions	1
Health Knowledge & Attitudes	3	Relationship to Person in Need of Care	7	Knowledge and Competences	0	Operational Efficiency	21
Patient Safety	11			Compliance	0	Financial Performance	4
				Physical/ Psychological Workload	2		
				Use of caregiving support	0		
Overarching Concepts		Overarching Concepts		Overarching Concepts		Overarching Concepts	
Well-being/ Quality of Life	17	Well-being/ Quality of Life	0			Hospital/Nursing Home Quality	4
Health-related QOL	13	Health-related QOL	0	Well-being/ Quality of Life	2		
Needs Assessment/ Fulfilled Needs	3	Caregiver Burden	0	Health-related QOL	0		
				Caregiver Burden	7		
Organisational		Organisational					
Intensity of Service Utilization	19	Employee Satisfaction	0				
Organisational Patient Safety Indicators	18	Professional Guideline Compliance	7				
Patient Satisfaction	6	Impact on Workload	2				
Intersectoral Continuity of Care	1						
Total number of studies*	77		20		10		45

*the total number of studies is lower than the amount (n) of the outcome areas from the above table, as single studies contain aspects from multiple outcome areas

For informal caregivers, caregiver burden is the most frequently analysed outcome area (*n* = 7), while all other outcome areas were rarely analysed or not at all (e.g. knowledge & competences *n* = 0; compliance *n* = 0; use of caregiving support *n* = 0). The most frequently analysed outcome areas for healthcare organisations are care process quality (*n* = 21), operational efficiency (*n* = 21) and communication/social interaction (*n* = 15). Little research has been done on financial performance (*n* = 4), access to care (*n* = 1) or working conditions (*n* = 1), while aspects of recruitment and staffing were not evaluated at all.

### Outcome areas of tools

This section provides a numerical analysis of all outcome tools included in the 123 articles. The numbers in Table 2 indicate the outcome tools used in each outcome area. The psychosocial state of persons in need of care was evaluated broadly using different tools (*n* = 69), as well as well-being/quality of life (*n* = 14), whereas social condition (*n* = 0) or health knowledge & attitudes (*n* = 0) were not analysed using tools. Formal caregivers were seldom examined using tools. Looking at the distribution of the available studies, psychological health (*n* = 3) was proportionally the most frequently measured outcome area for formal caregivers. Individual-related outcome areas such as medical condition or relationship to the person in need of care, or organisation-related individual outcomes such as employee satisfaction or impact on workload were not measured at all using outcome tools. Outcomes for informal caregivers were also rarely analysed using tools. The most frequently used concept was caregiver burden (*n* = 10). This is an overarching concept, which includes many of the single outcome areas listed for informal caregivers. A similar picture emerges for organisational factors. Only communication/social interaction (*n* = 4) and hospital/nursing home quality were evaluated using tools (*n* = 3). In the next sections, a detailed analysis is carried out to show which tools were used in the individual result areas.

**Table 2:** Numerical analysis of outcome tools

Persons in need of care	N	Formal Caregiver	N	Informal Caregiver	N	Healthcare Organisation	N
Functional Health	7	(Job) Satisfaction	1	(Life) Satisfaction	2	Care Process Quality	0
Physiological Health	9	Medical Health condition	0	Medical Health Condition		Access to Care	0
Psychological Health	69	Psychological Health	3	Psychological Health	2	Utilization of Services	0
Social Condition	0	Professional Knowledge and Competences	1	Social Condition	0	Communication/Social Interaction	4
Health Behaviour	1	Physical/ Psychological Workload	2	Living Conditions	0	Recruitment and Staffing	0
Health Knowledge & Attitudes	0	Relationship to Person in Need of Care	0	Knowledge and Competences	0	Working Conditions	0
Patient Safety	0			Compliance	0	Operational Efficiency	0
				Physical/ Psychological Workload	0	Financial Performance	0
				Use of caregiving support	0		
Overarching Concepts		Overarching Concepts		Overarching Concepts		Overarching Concepts	
Well-being/ Quality of Life	14	Well-being/ Quality of Life	0		0	Hospital/Nursing Home Quality	3
Health-related QOL	7	Health-related QOL	1	Well-being/ Quality of Life	0		
Needs Assessment/ Fulfilled Needs	3	Caregiver Burden	0	Health-related QOL	1		
				Caregiver Burden	10		
Organisational		Organisational					
Intensity of Service Utilization	1	Employee Satisfaction	0				
Organisational Patient Safety Indicators	2	Professional Guideline Compliance	0				
Patient Satisfaction	3	Impact on Workload	0				
Intersectoral Continuity of Care	0						

### Outcome tools used for specific target groups

#### Effectiveness relating to persons in need of care

In order to establish how past studies have attempted to measure the effectiveness of care technologies, we have listed all outcome tools that have been used and categorized them according to the outcome framework. Most of the studies included here investigated the effectiveness of a technology on persons in need of care (*n* = 77). Almost half of these studies (*n* = 38) used standardised instruments for measuring effectiveness. All instruments are listed in Tables 3 and 4 (for psychological measures) with a reference to the studies that use them. The corresponding outcome area is indicated in the top row of both Tables. A total of 69 different instruments were identified, most of which measure the psychological health of the person in need of care: 40 different instruments were used to measure the psychological health condition. The change effect is most frequently measured in terms of a state of depression, for example by using the Geriatric Depression Scale (*n* = 8) or the Cornell Scale for Symptoms of Depressions in Dementia (*n* = 4). The most frequently used single instrument for cognition is the Mini-Mental-State Examination (*n* = 8). In addition to mental state, the impact of technology on the quality of life was also frequently measured. The Quality of Life in Alzheimer’s Disease scale (QOL-AD) was most frequently used here (*n* = 7). Tools for the measurement of organisational patient safety indicators (*n* = 3) or intensity of service utilization (*n* = 3) were used less often. Most of the tools were only used in single studies.

**Table 3:** Outcome tools for person in need of care

N	Functional Health	N	Psychological Health	N	Organisational Patient Safety Indicators	N	Quality of Life	N	Health-related QOL	N	Needs Assessment/ Fulfilled Needs	N	Patient Satisfaction	N	Intensity of Service Utilization
2	IADL: Instrumental Activities of Daily Living Scale (Lawton) [47, 48]	1	BRADEN SCALE –For Predicting Pressure Sore Risk [49]	2	Agency for Health Research and Quality (AHRQ) Patient safety indicators [50, 51]	2	WHO-QOL: WHO Quality of Life Scale [21, 52]	2	EQ-5D (perceived level of health) [53, 54]	2	CANE: Camberwell Assessment of Need for the Elderly [21, 55]	2	HCAHPS: Hospital Consumer Assessments of Healthcare Providers and Systems [56, 57]	1	MCI: Medication Complexity Index [58]
1	GARS: Groningen Activity Restriction Scale [53]	1	PUSH Tool: Pressure Ulcer Scale for Healing [49]	1	AHRQ IQI: Inpatient Quality Indicators [51]	7	QOL-AD: Quality of Life - Alzheimer Disease [21, 55, 59–63]	2	SF-36: 36-Item Short Form Survey [58, 64]	1	IPPA: Individually Prioritized Problems Assessment score [12]	1	Frustration survey (Patak) [65]		
3	Barthel Index for Activities of Daily Living [54, 61, 64]	1	FACES: Wong Baker Faces Scale (Pain Measurement) [66]			3	QUALID-Scale: Quality of live in late-stage-dementia scale [67–69]	1	EQ-5D + c (perceived level of health and cognitive function) [55]						
1	ADL: Activities of Daily Living Scale (Lawton) [48]	1	PPT: Physical Performance Test [58]			1	ASCOT: Adult Social Care Outcomes Toolkit (well-being) [70]	1	SF-12: 12-Item Short Form Health Survey [71]						
1	MFES: Modified Falls Efficacy Scale [54]	1	Berg Balance Scale (physical performance) [64]					1	SF-8 Health Survey [72]						
								2	VAS: Visual analogue scale (pain intensity measure for adolescent self-report and caregiver observations [66, 73]						
								2	FLACC: Faces, Legs, Activity, Cry, Consolability) Pain Measurement [66, 73]

**Table 4:** Psychological health outcome tools for person in need of care

N	Tools	Measurement of:
1	COOP/WONCA Mood scale [12]	Mood
8	GDS: Geriatric Depression Scale [47, 48, 58–60, 62, 69, 71]	Depression
3	CSDD: Cornell Scale for Symptoms of Depressions in Dementia [63, 74, 75]	Depression
1	PHQ-9: Patient Health Questionnaire (Depression) [52]	Depression
1	HAM-D: Hamilton Depression Rating Scale [47]	Depression
1	BDI: Beck Depression Inventory [47]	Depression
1	GDS-12R: Geriatric Depression Scale (residential) [76]	Depression
4	CMAI/CMAI-SF: Cohen-Mansfield-Agitation Inventory Instrument [67, 74, 77, 78]	Agitation
2	Raid: Rating Anxiety in Dementia Scale [60, 75]	Anxiety
1	BARS: Brief Agitation Rating Scale [77]	Agitation
1	BAI: Beck Anxiety Inventory [47]	Anxiety
1	Burn Specific Pain Anxiety Scale (BSPAS) [79]	Anxiety
1	AOL: Alertness Observation (check)-List [80]	Alertness
1	PSS: Perceived Stress Scale [47]	Stress
1	RAWS: Revised Algase Wandering Scales [60]	Wandering
1	APADEM-NH: Apathy scale [69]	Apathy
1	AI: Apathy Inventory [69]	Apathy
1	AES: Apathy Evaluation Scale [60]	Apathy
1	GSR: Galvanic skin Response (measuring emotional arousal) [75]	Affect
1	OERS: Observed Emotion Rating Scale [60]	Affect
2	UCLA: loneliness scale [52, 62]	Loneliness
1	DJGLS: De Jong Gierveld Loneliness Scale [81]	Loneliness
4	NPI: Neuropsychiatric Inventory [47, 55, 61, 69]	Psychological Symptoms
1	OQ-45 -questionnaire (psychological patient progress) [52]	Psychological Symptoms
1	BNT: Boston Naming Test [48]	Psychological Symptoms
1	NPI-Q: Neuropsychiatric Inventory Questionnaire [74]	Psychological Symptoms
1	Pearlin Mastery Scale (psychological resources) [21]	Psychological Resources
8	MMSE: Mini-Mental State Examination [21, 47, 48, 58, 61, 63, 64, 69]	Cognition
2	TMT: Trail Making Test A/B (Visual attention and task switching) [47] [48]	Cognition
1	sMMSE: Severe Mini Mental State Examination [69]	Cognition
1	MoCA: Montreal Cognitive Assessment [47]	Cognition
1	FUCAS: Functional Cognitive Assessment Scale [47]	Cognition
1	CDT: Clock Drawing Test (cognitive impairment) [48]	Cognition
1	GDS* Global Deterioration Scale (cognitive function) [75]	Cognition
1	ACE-R: Addenbrooke’s Cognitive Examination-Revised [82]	Cognition
2	ROCF: Rey–Osterrieth complex figure (spatial visual construction and visual memory) [47, 48]	Memory
2	RAVLT: Rey Auditory Verbal Learning Test [47, 48]	Memory
1	RBMT: Rivermead Behavioral Memory Test [47]	Memory
1	Digit Span Memory Test [48]	Memory
1	TEA: Test of Everyday Attention [47]	Attention

#### Effectiveness relating to caregiver

Significantly fewer studies from our sample relate to results for caregivers. Only 30 studies referred to caregiver outcomes, and eleven of them used standardised instruments (Table 5). A total of 20 different instruments were found, four of them specifically for formal caregivers and, ten for informal caregivers; five are universal instruments. Though there are fewer studies on informal caregivers (*n* = 10) than on formal caregivers (*n* = 20), the informal caregiver burden was the most frequently addressed outcome area for outcome tools. The second most frequently used category of tools addresses psychological changes and the third most frequently used category of instruments evaluates changes in satisfaction. Instruments for QOL, knowledge and workload were the least used. We did not find any instruments for measuring the physical load of caregivers. No tool can be named that has been used particularly frequently for the evaluation of caregivers. Almost all tools were used only once in the sample.

**Table 5:** Caregiver relevant outcome tools

n	Psychological Health	n	Caregiver Burden	n	(Job) Satisfaction	n	Health-related QOL	n	Professional Knowledge and Competences	n	Impact on Workload
**1**	PERI-D: Psychiatric Epidemiology Research Instrument^2^ (Demoralization Scale) [59]	2	SSCQ: Short Sense of Competence questionnaire^2^ (dealing with burden) [21, 55]	1	Job Satisfaction Score^1^ (Hagopian et al.) [59]	1	SF-12: 12-Item Short Form Health Survey^1^ [59]	1	Palliative and End of Life Care competency Assessment Tool^1^ [83]	1	NASA-TLX: The NASA Task Load Index^1^ [84]
**1**	MM-CGI: Marwit Meuser Caregiver Grief Inventor – short form^2^ [85]	2	ZBI-12: Zarit Burden Interview - short form^2^ [85, 86]	1	CSS: Caregiving Satisfaction Scale^2^ [86]	1	EQ-5D + c (perceived level of health and cognitive function)^2^ [55]			1	RUD-FOCA: Resource Utilization in Dementia – Formal Care ^1^ [63]
**1**	PHQ-9: Patient Health Questionnaire ^2^ (Depression) [85]	1	DIS: Desire to Institutionalize Scale^2^ [85]	1	Press-Ganey™ patient satisfaction surveys^2^ [87]						
**1**	NPI: Neuropsychiatric Inventory^2^ [55]	1	NAC: National Alliance for Caregiving survey^2^ [86]								
		1	CSI: Caregiver Strain Index^2^ [53, 86]								
		1	SPPIC: Self- Perceived Pressure from Informal Care – Scale^2^ [88]								
		1	OBM: Objective Burden Informal Caregiver’^2^ [53]								
		1	SRB: Self Rated Burden ^2^ [53]								

1: used for formal caregivers; 2: used for informal caregivers

#### Organisational effectiveness

Aspects of organisational effectiveness were measured in 45 studies. Seven studies used different standardised instruments. We found four instruments to analyse communication/social interaction in the respective organisation and three instruments to analyse hospital quality (Table 6). Each tool was used only once in the sample.

**Table 6:** Organisation-related outcome tools

n	Hospital/Nursing Home Quality	N	Communication/Social Interaction
1	QAS: Quality Improvement Activities Survey [57]	1	Perception of Communication Difficulty Questionnaire [65]
1	CPS: Clinicians’ Perceptions of Quality Survey [57]	1	Frustration with Communication tool [65]
1	CalNOC: Medical Administration Accuracy Observation Codesheet [89]	1	CSACD: Formal Caregiver: Collaboration and Satisfaction About Care Decisions survey instrument [90]
		1	QCPR: quality of caregiving relationship [76]

## Discussion

The aims of this scoping review are (i) to show which possible outcome areas for measuring effectiveness of DNT can be identified, (ii) to depict which areas have been the focal point of research on effectiveness of DNT so far, and which areas have been researched less, and (iii) to show how effectiveness has been measured in previous studies. The discussion section is structured around these research questions. Therefore, we divided the discussion section into three main parts: discussion of the framework development (i), critical reflection in relation to the scientific literature (i) and discussion of the quantitative analysis (ii & iii).

### Framework development

The comprehensive DNT outcome framework was developed to show which possible outcome areas can be identified for the evaluation of DNT. This framework can be used by researchers to structure their effectiveness evaluation and to check whether essential outcome areas are considered in their evaluation. Thus, its purpose is thus to encourage researchers to focus on specific outcome areas or include additional outcome areas in their work. It is also intended to promote and structure discussion and reflection on desirable or necessary research objectives of DNT and may help to draw inferences on areas in which undesirable negative effects may emerge. The framework was developed using deductive and inductive methods, and therefore comprises elements that have already been researched specifically for the field of technology as well as a derivation from general nursing contexts to the specific context of DNT. Parts of the framework are therefore generic and could also be used for the evaluation of general nursing care interventions. We have also included both effectiveness and efficacy studies in the development of the Framework. Being aware of the differences of these two concepts, we assume that the incoherent use of the words within the analysed studies was caused by the fact that “efficacy and effectiveness exist on a continuum” [91] and the generalizability depends on the viewpoint of the observer and the observed condition [91]. The incorrect or incoherent classification of the two terms has already been described in the scientific literature [92]. In order to allow researchers to choose from a variety of possible outcomes adapted to the particular circumstances and context of the study, and to decide which outcome areas are to be evaluated, it is necessary to develop the most comprehensive framework possible. This led us to include studies referring to themselves as effectiveness studies and studies that refer to themselves as efficacy studies. We leave it to the judgement of the respective researchers to decide which are the optimal outcome areas and corresponding outcomes for their aspired studies.

### Critical reflection of the framework in relation to the scientific literature

When comparing our framework with existing frameworks in the field of digital technologies in health care we can state that to the best of our knowledge there is no other such comprehensive framework with a special focus on nursing. As stated in the methods section, we incorporated other frameworks in the development of the DNT framework. To highlight the specific strengths of the newly developed framework, we shall now briefly describe the differences between the DNT framework and some other frameworks in similar contexts. The most comprehensive framework available (MAST) was incorporated in the design of the DNT framework [18]. MAST provides seven domains, including a focus on patients and a focus on the organisation. Five of the seven domains are highly relevant to the nursing context and were therefore included in the development of the DNT outcome framework (safety, clinical effectiveness, patient perspectives, economic aspects, organisational aspects). Formal caregivers are only rarely considered in MAST, and informal caregivers are not considered at all. These target groups are presented and highlighted in much greater detail in the DNT results framework. As a holistic framework, MAST also contains references to socio-cultural, ethical and legal aspects. These are important areas for the evaluation of DNT in general, but they do not fit into the specific context of an effectiveness evaluation for DNT that we wanted to depict in this study. The analysis of ethical and socio-cultural effects requires different research approaches, which are not reflected in our sample. In order to cover these areas, it would be necessary to include a macro perspective underpinned by scientifically sound data. We have deliberately not focused on the macro level, but it would be a possibility to complement this with further research.

For a second comparison, the Infoway Benefits Evaluation Framework [17] is used. This framework divides the evaluation of health information systems into six main dimensions: system, information, service, use, satisfaction and net benefits. The “net benefits” dimension includes many outcome areas such as patient safety, health outcomes, access to care and productivity that were integrated into the DNT outcome framework. User (in this case patient) satisfaction was also integrated into the DNT outcome Framework, but we added areas relating to formal and informal carers, as they were not taken sufficiently into account in the Infoway Benefits Evaluation Framework. Other attributes such as accuracy, performance or functionality refer primarily to the effectiveness of the respective technology, so we therefore excluded them from the DNT Framework.

For another comparison we refer to the adapted version of the Nursing Care Performance Framework [2], which displays the impact of ICT solutions on nursing care. This framework shows important areas especially for formal caregiving, which can also be found in the DNT Outcome Framework. Informal caregivers are not represented, which is certainly due to the focus. The effects on patients are presented in a very specific way. Our DNT outcome frameworks can be helpful to complement some details on outcome areas, such as psychological health or health behaviour. The comparison of the DNT framework with the systematization in a systematic review of effectiveness studies in the field telemedicine [93], indicates that the DNT Framework covers all important outcome areas on the micro and meso level. Ekelanda et al. also include a few aspects on the macro level, e.g. in the area of politics [93]. This level was excluded in the DNT outcome framework, as it implies a different perspective of analysis, and none of the studies in the scoping review related to the macro level. The exploration of outcome areas, outcome tools and outcome measures on the macro level, however, is an interesting field for future research. In sum, it can be said that the developed DNT outcome framework closes an existing gap in nursing and technology research by including all important outcome areas relevant to nursing.

### Reflection on the included outcome areas and outcome tools

We evaluated all outcome tools and measures of the included 123 articles with a view to establishing which outcome areas have so far been focused on by research on DNT effectiveness, and which areas have been researched less. There are considerable differences in the researched outcome areas for the individual outcome target groups. It should be pointed out, however, that no valuation of the significance of an outcome area can be made at present. This could be the topic of further research. The study presented here constitutes a first step towards summarizing existing trends.

Persons in need of care were by far the most frequently researched target group. Psychological health, intensity of service utilization, and organisational patient safety indicators were measured much more frequently than needs assessment/ fulfilled needs, health knowledge & attitudes or intersectoral continuity of care. There might be several reasons for this. On the one hand, it might be more difficult to capture fulfilled needs, intersectoral continuity of care, or health knowledge & attitudes with the existing standardised instruments or outcome measures. On the other hand, it might simply not have been of such profound interest during the evaluation because the respective technologies did not target these areas. It is interesting to note that intersectoral care is an area that has so far largely been neglected. Intersectoral care in form of communication or collaboration between different healthcare sectors (e.g. ambulatory care and inpatient care) [94] could, for example, help to prevent hospitalisations [95]. One reason for the neglection of intersectoral care might be that while it is already difficult to implement digital technologies in a single sector, sector boundaries possible constitute a major challenge. The decision-making structures of a healthcare system might be seen to be a barrier to change in this context [96].

Overall in our sample, formal and informal caregivers have not been researched frequently in terms of the effectiveness of DNT. Relationship to person in need of care was most frequently measured among formal caregivers, along with guideline compliance and physical/psychological workload. Well-being/quality of life, health-related quality of life, medical condition and caregiver burden were not measured once in our sample. Several other outcome areas were very rarely analysed. This shows that these aspects were neglected in the past, while quality of life and caregiver burden on professional caregivers are still not being evaluated. The reasons for this should be clarified. It is unclear whether this is the case because these outcome areas are generally considered to be less important than others, or whether there are other reasons for non-evaluation. Assuming that an important goal of digital technologies in nursing care is to relieve nursing staff, it seems inappropriate to only evaluate the direct workload (e.g. physical load or psychological stress through direct work) and not evaluate the effects of digital technologies on the general burden on or the quality of life of formal caregivers. Existing instruments such as the Professional Care Team Burden (PCTB) scale harbour the potential to contribute to the evaluation of DNT in this context [97].

The caregiver burden of informal caregivers was the most frequently analysed outcome area, while all other outcome areas were analysed very seldom or not at all. One reason might be that most instruments for measuring caregiver burden have been developed for informal caregivers [97]. On the other hand, there seems to have been a socio-political interest in reducing the burden on family members through technology in the past. At the same time, the medical health condition, knowledge and competences or the use of caregiving support of informal caregivers have not been analysed in a single study. Medical health may not have been recorded for reasons of personal data protection. Specialised nursing knowledge and skills do not seem to play such a large role in research on technological support for informal carers. We expect enhanced support for informal caregivers to play a more important role in the future, and, hence, those research areas that are seldom examined now to become more important.

The most frequently analysed outcome areas for healthcare organisations were care process quality, operational efficiency and communication/social interaction. Little research has been done on financial performance, access to care and working conditions, while recruitment and staffing areas were not evaluated at all. The frequently researched areas correspond with the potential goal of technologies to improve efficiency while maintaining a high quality of care [98]. Aspects of working conditions or effects on the recruitment or staffing processes from an organisational perspective have apparently never been analysed. If it is assumed that, from an organisational point of view, the main purpose of the technologies is to increase efficiency while maintaining or improving the quality of care, factors such as working conditions for carers play a minor role. This is consistent with the results on formal caregivers. Nevertheless, it is interesting that the impact of existing technologies on recruitment has not been investigated, as technologies are often claimed to be beneficial in terms of enhancing the attractiveness of a healthcare organisation for prospective and current employees [99], which could be expressed in an evaluation of the effects of a technology on recruitment figures. In the light of the current lack of skilled workers, proof of such effects might be an interesting finding.

The third research question was addressed by showing which outcome areas were evaluated with outcome tools and which areas were more likely to be covered by other measures. At the same time, the variety and range of the tools used were presented. The wide range of outcome tools – especially in the field of psychological health - makes it difficult to compare the studies’ results, and a common set of standards for using outcome tools shared by several studies would considerably help improve comparability To this end, further research is needed to assess and evaluate existing outcome tools.

### Limitations

Limitations that refer to the underlying scoping review are described in Krick et al. 2019. Especially important for this additional methodological analysis are the following aspects:

Publication bias in particular should be considered here. Studies without positive results are often not published in journals. Outcome areas for which effects are difficult to prove may be underrepresented in this article due to this publication bias, whereby, there is a possibility that studies on certain technologies may have been over-represented, under-represented or not presented at all due to negative results. This may indirectly affect the presented outcome tools and measures.

The included outcome areas and outcome tools presented could also be influenced by the fact that we included both effectiveness and efficacy studies in the development of the framework. It could be criticised that a further breakdown of a framework for efficacy studies and a framework for effectiveness studies is necessary because they may differ in their outcomes and tools. However, we decided to combine these concepts for the reasons of applicability and comprehensiveness, as described in the discussion section. Based on the included publication period of 7 years, the question needs to be considered whether outcome tools or outcome measures are only mapped for the indicated period, and therefore relevant measures of the effectiveness of DNT applied outside that period are missing. There is also the possibility that outcome areas overlap or might have been composed differently by other researchers. Overall, we have tried to ensure the highest possible standard for the outcome framework, by combining literature and expert knowledge. The current version is very comprehensive, but the field of research on DNT is very dynamic, and future adaptions should be included.

## Conclusion

This scoping review provides a broad overview on outcome areas and outcome tools used for the evaluation of digital nursing technologies. All outcome tools and measures have been categorised according to our newly developed DNT outcome framework to show which areas have been focused on by research on effectiveness of DNT so far and which areas have rather been neglected. We highly recommend the use of this framework (and the further explanations given in the Additional files) as a basis for future research. Researchers can use the DNT outcome framework as a tool to structure their effectiveness evaluations and to examine whether essential outcome areas have been overlooked in their evaluations. Currently, the DNT outcome framework mainly provides an overview of all outcome areas. The weighting of the importance or significance of the different outcome areas – especially those that have been less explored so far – should be subjected to further research. This would require a more detailed assessment of the individual outcome areas, including a valuation of the areas in the subsequent research. We also consider it important to investigate the heterogeneity of interventions in DNT and to deepen the understanding of different important outcomes linked to these DNTs.

Our systematized overview of the tools in the individual areas can be used as a starting point for further research, in order to share and compare information about appropriate tools and initiate a discussion on the standardisation of tools used for similar questions. An appropriate exchange would also certainly be helpful for outcomes measures used that are not listed here in detail due to the large number.

So far, our findings indicate that intersectoral continuity of care for persons in need of care, quality of life of formal and informal caregivers, caregiving support for informal caregivers and working conditions from an organisational perspective are outcome areas which have only been scantily researched so far and would benefit considerably from future research. At the same time, we recommend the development of outcome areas for the macro level of effectiveness assessment, which is not included in the current version of the DNT outcome framework. Overall, the DNT outcome framework already offers a very good overview of the possible outcome areas and we are confident that future research will benefit from this structured approach.

## Supplementary information

**Additional file 1.** Definitions and Examples for Outcome Areas for Persons in Need of Care.

**Additional file 2.** Definitions and Examples for Outcome Areas for Outcome Areas Formal Caregivers.

**Additional file 3.** Definitions and Examples for Outcome Areas for Outcome Areas Informal Caregivers.

**Additional file 4.** Definitions and Examples for Outcome Areas for Outcome Areas Organisation.

**Additional file 5.** Included Technologies.

## Data Availability

The datasets used and/or analysed during the current study are available from the corresponding author on reasonable request.

## Abbreviations

ACM: Association for Computing Machinery
DNT: Digital Nursing Technologies
MAST: Model for Assessment of Telemedicine
NOC: Nursing Outcomes Classification
QOL: Quality of life
QOL-AD: Quality of Life - Alzheimer Disease
