# Supplementary material for: Measuring the effectiveness of digital nursing technologies: development of a comprehensive digital nursing technology outcome framework based on a scoping review
Source: BMC Health Serv Res. 2020 Mar 24;20:243. doi: 10.1186/s12913-020-05106-8 (PMC7092516; doi:10.1186/s12913-020-05106-8)
Supplement: Supplementary file 1 — Additional file 1. Definitions and Examples for Outcome Areas for Persons in Need of Care. [file 12913_2020_5106_MOESM1_ESM.pdf]

Effectiveness Outcomes – Person in need of care related

**Outcome Area**	**Examples for Subcategories or Indicators**
**Functional Health**<br>Outcomes that describe capacity for and performance of basic tasks of life*	- Mobility<br>- Self-care (ADL/IADL)<br>- Energy maintenance (i.e. sleep patterns)<br>- Activity Level<br>- Required care levels<br>- Level of independence (perceived)
**Physiological Health**<br>Outcome that describe organic functioning*	- Metabolic Regulation (vital signs)<br>- Electrolyte & Acid/Base Balance<br>- Tissue Integrity (i.e. wound healing)<br>- Neurocognitive functions/condition<br>- Therapeutic response (reactions to treatment)
**Psychological Health**<br>Outcomes that describe psychological functioning*	- Psychological well-being (i.e. agitation, loneliness, stress-level)<br>- Psychosocial adaption (i.e. Coping, acceptance of health status)<br>- Self-control (challenging behaviour)
**Social Condition**<br>Outcomes that describe social functioning and social inclusion	- social interactions (verbal and visual engagement)<br>- social relation to caregiver<br>- social participation/ social inclusion
**Health Behaviour**<br>Outcomes that describe an individual's actions to promote or restore health*	- Therapy compliance<br>- Dealing independently with requirements and strains caused by illness or therapy<br>- Patient engagement
**Health Knowledge & Attitudes**<br>Outcomes that describe an individual's understanding in applying information to promote maintain and restore health* (or an individual's ideas and perceptions that influence health behaviour*)	- Knowledge on health promotion, healthy lifestyle<br>- Knowledge on medication (pain management)<br>- Disease/health situation related knowledge<br>- Perceived ability to perform
**Patient Safety**<br>Outcomes that describe an individual's safety status*	- Fall prevention behaviour<br>- Falls occurrence
**Overarching Concepts** (Comprise different aspects of the above-mentioned dimensions)	
**Well-being/ Quality of Life**<br>Outcomes that describe an individual's perceived health status and life circumstances*	- General QoL-Indicators<br>- Disease specific QoL-Indicators<br>- Subgroup specific QoL-Indicators<br>Indicators<br>- ASCOT (Adult Social Care Outcomes Toolkit)<br>- WHO-QoL (WHO-Quality of Life Scale)<br>- QoL-AD (Quality of Life in Alzheimer Disease)
**Health related QoL**<br>Outcomes that refer to a valuation of an individual's perceived physical, mental and (optionally) social well-being	- General self-perceived health status<br>- Disease or symptom specific self-perceived health status (i.e. pain level, symptom severity)<br>Indicators<br>- EQ-5D, SF-36

**Needs Assessment/ Fulfilled Needs**<br>Outcomes that relate to systematic processes for determining and addressing needs (discrepancies between current and wanted conditions)	Indicators<br>- IPPA (Individually Prioritized Assessment score)<br>- CANE (Camberwell Assessment of Need for the Elderly

Individual-related organisational Effectiveness

**Intensity of Service Utilization**<br>Outcomes that describe the length or intensity of usage of a specific health care service	- Length of stay<br>- Number of (re)admissions to specific institutions<br>- Days in need of specific services/medication (i.e. number of days with antibiotics)
**Organisational Patient Safety Indicators**<br>Outcomes that describe safety-related negative organisational incidents	- Mortality<br>- Occurrence of medical complications (i.e. number of cardiac arrest calls, infection rates)<br>- Occurrence of adverse events (i.e. falls)
**Patient Satisfaction**<br>Outcomes that describe an individual's perception of the quality and adequacy of health/nursing care provided*	- Satisfaction with access to care resources<br>- Satisfaction with performance of services<br>- Satisfaction with adequacy of provided services
**Intersectoral Continuity of Care**<br>Outcomes relating to patient transfers and the collaboration between different care providers or different units of care providers	- Continuity of care inside institutions (i.e. different units in hospitals)<br>- Continuity of care between different institutions

*** Definitions based on NOC (5th edition)**

5th Edition:

https://www.academia.edu/38156239/Nursing_Outcomes_Classification_NOC_Moorhead_HERRY_.pdf
