# Supplementary material for: Measuring the effectiveness of digital nursing technologies: development of a comprehensive digital nursing technology outcome framework based on a scoping review
Source: BMC Health Serv Res. 2020 Mar 24;20:243. doi: 10.1186/s12913-020-05106-8 (PMC7092516; doi:10.1186/s12913-020-05106-8)
Supplement: Supplementary file 2 — Additional file 2. Definitions and Examples for Outcome Areas for Outcome Areas Formal Caregivers. [file 12913_2020_5106_MOESM2_ESM.pdf]

Effectiveness Outcomes – Formal Caregivers

**Outcome Area**	**Examples for Subcategories or Indicators**
**(Job) Satisfaction**<br>Outcomes that relate to the subjective evaluation of the current life or work situation/condition by a person him/herself	- General job satisfaction<br>- Life satisfaction<br>- Satisfaction with flexibility and autonomy<br>- Satisfaction with professional status
**Medical Health condition**<br>Outcomes that describe the health situation of the formal caregiver	- Occurring diseases<br>- Symptoms (i.e. pain, fatigue)<br>- Use of healthcare services
**Psychological Health**<br>Outcomes that describe psychological functioning*	- Psychological well-being (self-confidence, stress, depression loneliness …)<br>- Psychosocial Adaption (i.e. coping)
**Professional Knowledge and Competences**<br>Outcomes that indicate the level of existing professional knowledge, skills and behavioural patterns necessary for the practice of professional nursing	- Knowledge on safety regulations (regarding patients)<br>- Knowledge on disease related care<br>- Knowledge on occupational safety<br>- Professionalism
**Physical/ Psychological Workload**<br>Outcomes that describe the physical and/or mental effort during the performance of a specific care activity	- Physical Workload (incl. tasks)<br>○ Force required to handle Patient<br>○ Walking distance<br>- Psychological Load<br>○ Stress in specific work situations
**Relationship to the Person in Need of Care**<br>Outcomes that indicate the degree of personal and professional connection to the patient	- Relationship continuity<br>- Empathic resonance<br>- Trust<br>- Interpersonal treatment<br>- Time with patient (for building a relationship)
**Overarching Concepts** (Comprise different aspects of the above-mentioned dimensions)	
**Well-being/ Quality of Life:**<br>Outcomes that describe an individual's perceived health status and life circumstances*	- General QoL-Indicators<br>- Disease specific QoL-Indicators<br>- Subgroup specific QoL-Indicators<br><br>Indicator<br>- WHO-QoL (WHO-Quality of Life Scale)
**Health related QoL**<br>Outcomes that refer to a valuation of an individual's perceived physical, mental and (optionally) social well-being	- General self perceived health status<br>- Disease or symptom specific self perceived health status (i.e. pain level, symptom severity)<br>- Indicators<br>- EQ-5D: EuroQol-5D<br>- SF-36: 36-Item Short Form Health Survey
**Caregiver Burden**	

Multifactorial outcomes on work related burdens that a professional caregiver is exposed to	Indicators<br>- Professional Care Team Burden (PCTB), Caregiver Burden Inventory (CBI)

Individual-related organisational Effectiveness

**Outcome Area**	**Examples for Subcategories or Indicators**
**Employee Satisfaction**<br>Outcomes that indicate the well-being and general satisfaction with respect to the working conditions of all employees in an organizational unit	- Staff moral<br>- Absenteeism<br>- Attrition
**Professional Guideline Compliance**<br>Outcomes that indicate the degree to which formal caregivers act according to professional guidelines	- Self-monitoring compliance<br>- Documented compliance<br>- Compliance awareness<br>- Access to guideline information
**Impact on Workload**<br>Outcomes that describe the functional load that the performance of a particular work activity of a professional caregiver implies, when it is considered at the organizational level	- Workload<br>○ Tasks<br>○ Frequency<br>○ Intensity (daily work hours)<br>○ Time spent on caregiving

*** Definitions based on NOC (5th edition)**

5th Edition:

https://www.academia.edu/38156239/Nursing_Outcomes_Classification_NOC_Moorhead_HERRY_.pdf
