# Supplementary material for: Measuring the effectiveness of digital nursing technologies: development of a comprehensive digital nursing technology outcome framework based on a scoping review
Source: BMC Health Serv Res. 2020 Mar 24;20:243. doi: 10.1186/s12913-020-05106-8 (PMC7092516; doi:10.1186/s12913-020-05106-8)
Supplement: Supplementary file 3 — Additional file 3. Definitions and Examples for Outcome Areas for Outcome Areas Informal Caregivers. [file 12913_2020_5106_MOESM3_ESM.pdf]

Effectiveness Outcomes –Informal Caregiver

**Outcome Area**	**Examples for Subcategories or Indicators**
**(Life) Satisfaction**<br>Outcomes that relate to the subjective evaluation of the current life or care situation/condition by a person him/herself.	- Satisfaction with life situation<br>- Satisfaction with care situation<br>- Satisfaction with care services
**Medical Health Condition**<br>Outcomes that describe the health situation of the informal caregiver	- Use of healthcare services<br>- Symptoms (i.e. pain, fatigue)<br>- Occurring diseases
**Psychological Health**<br>Outcomes that describe psychological functioning*	- Psychological well-being (i.e. depression, loneliness, stress-level)<br>- Psychosocial adaption (i.e. Coping, acceptance of care situation)
**Social Condition**<br>Outcomes that describe social functioning and social inclusion	- Social relation to care-receiver<br>- Social participation/ social inclusion<br>- (Social relation to family/significant others)<br>- Family Health (health status, behaviour or functioning of the family, relationship to the patient)
**Living Conditions**<br>Outcomes that describe the effects of the care situation on general life circumstances of a caregiving person	- Daily Activities<br>- Work Life<br>- Family well-being<br>- Financial Situation
**Knowledge and Competences**<br>Outcomes that describe a caregiver's understanding in applying information to promote maintain and restore health,* and to support and care for the care-receiver	- Knowledge on health promotion, healthy lifestyle<br>- Disease/care situation related knowledge<br>- Knowledge on supportive infrastructures?
**Compliance**<br>Outcomes that describe the degree to which informal caregivers comply with professional advices	- Compliance awareness<br>- Access to guideline information<br>- Self-monitored compliance
**Physical/ Psychological Workload:**<br>Outcomes that describe the physical and/or psychological effort during the performance of a specific care activity	- Physical Workload (incl. tasks)<br>- Psychological Workload<br>- Time spent on caregiving<br>- Care performance
**Use of caregiving support**<br>Outcomes that describe the amount of additional informal or professional care support that is needed in the care situation	- Use of professional care services<br>- Use of non-professional care support resources (i.e. self-help groups)
**Overarching Concepts** (Comprise different aspects of the above-mentioned dimensions)	
**Well-being/ Quality of Life**<br>Outcomes that describe an individual's perceived health status and life circumstances*	- General QoL-Indicators<br>- Subgroup specific QoL-Indicators
**Health related QoL**<br>Outcomes that refer to a valuation of an individual's perceived physical, mental and (optionally) social well-being	- General self-perceived health status<br>- Disease or symptom specific self-perceived health status (i.e. pain level, symptom severity)<br>Indicators

	- EQ-5D: EuroQol-5D<br>- SF-36: 36-Item Short Form Health Survey
**Caregiver Burden** Outcomes that describe the strain perceived by caregivers due to a home care situation (caused by caregiving activities and responsibilities)	Indicators<br>- ZBI-12: Zarit Burden Interview-short form<br>- CSI: Caregiver Strain Index<br>- OBM: Objective Burden Informal Caregiver'

*** Definitions based on NOC (5th edition)**

5th Edition:

https://www.academia.edu/38156239/Nursing_Outcomes_Classification_NOC_Moorhead_HERRY_.pdf
