# Supplementary material for: Measuring the effectiveness of digital nursing technologies: development of a comprehensive digital nursing technology outcome framework based on a scoping review
Source: BMC Health Serv Res. 2020 Mar 24;20:243. doi: 10.1186/s12913-020-05106-8 (PMC7092516; doi:10.1186/s12913-020-05106-8)
Supplement: Supplementary file 4 — Additional file 4. Definitions and Examples for Outcome Areas for Outcome Areas Organisation. [file 12913_2020_5106_MOESM4_ESM.pdf]

Effectiveness Outcomes – Organisation

Outcome Area	Examples for Subcategories or Indicators
**Care process quality**<br>Outcomes that indicate the general quality of the nursing process in an organisation	- Risk of poor treatment<br>- Quality of assessment and documentation<br>- Healing/Recovery rates<br>- Readmission rates<br>- Medication administration (errors, accuracy)
**Access to care**<br>Outcomes that describe the degree of the access to and availability of certain services	- Accessibility (physical/remote)<br>- Availability of services (geographic, timeliness, barriers)
**Communication/Social interaction**<br>Outcomes that describe the communication between different groups within an organization and the social interactions and collaboration	- Relationship quality<br>- Communication intensity<br>- Communication errors<br>- Interprofessional relationship
**Recruitment and Staffing**<br>Outcomes that describe aspects related to the recruitment of employees and the maintenance of employee quantity and quality	- Recruitment related outcomes<br>- Staff retention<br>- Absenteeism<br>- Personnel development
**Working Conditions**<br>Outcomes that describe the general working conditions within an organisation	- Physical (noise, vibration)<br>- Organisational (monotonicity, variety)<br>- Working Hours (nightshifts, scheduling)
**Operational Efficiency**<br>Outcome measures that indicate the degree to which efficient processes are designed by an organization to help improve productivity and performance	- Efficient usage of resources<br>- Organisational workflow<br>- Operational efficiency (staff per patient)<br>- Efficiency of work processes<br>- Productivity
**Financial Performance**<br>Outcomes that indicate the financial result that an organization is capable of producing	- Profitability<br>- Financial sustainability<br>- Debts
**Overarching Concepts** (Comprise different aspects of the above-mentioned dimensions)	
**Hospital/Nursing Home Quality**<br>Outcomes that summarise the quality of a care of an institution in different dimensions	Indicators:<br>- QAS: Quality Improvement Activities Survey<br>- CPS: Clinicians' Perceptions of Quality Survey
