# Supplementary material for: Measuring the effectiveness of digital nursing technologies: development of a comprehensive digital nursing technology outcome framework based on a scoping review
Source: BMC Health Serv Res. 2020 Mar 24;20:243. doi: 10.1186/s12913-020-05106-8 (PMC7092516; doi:10.1186/s12913-020-05106-8)
Supplement: Supplementary file 5 — Additional file 5. Included Technologies. [file 12913_2020_5106_MOESM5_ESM.pdf]

Additional File 5: Included Technologies

Technology subcategory / Specific technology	Authors/Year
**1. Studies on ICT**	
**1.1. Studies on Health Institution Information System (HIS)**	
HIS (hospital)	(Angst et al. 2012), (Appari et al. 2014), (McKenna et al. 2017), (Restuccia et al. 2012)
HIS (ICU)	(Steurbaut et al. 2012)
HIS (nursing home)	(Alexander et al. 2014), (Alexander et al. 2015), (Munyisia et al. 2012)
HIS (subsystem/ patient engagement)	(Patmon et al. 2016)
**1.2. Studies on Electronic Health Records/Electronic Medical Records**	
EMR in long-term care	(Hitt & Tambe 2016), (Meehan 2017), (Rantz et al. 2011)
EMR in hospitals in general	(Mitchell & Yaylacicegi 2012), (Bradley 2011), (Takian et al. 2012), (Yusof 2015)
Decision support/Data results management	(Lo et al. 2014)
Medication Administration	(Seibert et al. 2014), (Appari et al. 2012), (Chanyagorn et al. 2016), (Ching et al. 2014), (Huang & Lee 2011)
Patient handoff/health information exchange	(Clarke et al. 2017), (Oakley & Hunter 2017), (Yeaman et al. 2015), (Meyer-Delpho & Schubert 2014)
Patient information administration/Nurse reminding system	(Lear & Walters 2015), (Paranilam 2013)
**1.3. Studies on Computerised Decision Support Systems**	
Risk assessment	(Lapane et al. 2011), (Dykes et al. 2012)
Care Decisions	(Lang 2012), (Salinas et al. 2011)
**1.4. Studies on Telecare**	
Video-Telecare	(van der Heide et al. 2012), (Cady 2012), (Cady & Finkelstein 2014)
Video Telecare incl. remote monitoring	(Bowles et al. 2011)
Remote health-monitoring	(Steventon et al. 2013), (Wakefield & Vaughan-Sarrazin 2017) (Paré et al. 2013)
Telecare per Instant-Messaging	(Chiang & Wang 2016)
Telecare/ App supported	(Göransson et al. 2017)
Telecare/ Internet- vs. telephone-based support	(Hicken et al. 2017)
**1.5. Studies on Communication Support Technologies**	
**1.5.1. Communication Support between professionals**	
Cloud based smartphone nurse-call system	(Chuang et al. 2015)
Hands free communication	(Pemmassani et al. 2014)
Discharge huddle with mobile technology	(Tielbur et al. 2015)
Tele-conferencing for remote training of health care providers	(White et al. 2015)
Wireless call handling and task management system (out of hours)	(Blakey et al. 2012)
Hospital-home care collaboration by electronic messaging	(Melby et al. 2015)
Smartphone use in clinical communication	(Wu et al. 2011)
**1.5.2. Communication support between professionals and patient/relatives**	
Communication between formal caregiver and patient/ for suddenly speechless patients	(Rodriguez 2016)
Communication between professionals and relatives /intraoperative communication	(Wieck et al. 2017)
**1.6. Studies on Specific Software/Apps**	
**1.6.1. Care support for professionals**	
Provision of informa-tion about residents	(Webster & Hanson 2014)
Point of care documentation	(Yi-Sheng et al. 2014)
Point of care wound documentation	(Florczak et al. 2012)
Wound monitoring and remote support	(Vowden & Vowden 2013)

**1.6.2. Care support for informal caregivers**	
Dementia specific digital social chart	(Mierlo et al. 2015)
**1.6.3. Patient support for everyday life**	
Personal assistant for dementia	(Nijhof et al. 2013a)
**1.6.4. Therapeutic support for patients/persons in need of care**	
Cognitive stimulation	(Zaccarelli et al. 2013), (Zhuang et al. 2013), (Berenbaum et al. 2011), (Nordheim et al. 2015)
Digital life story books	(Subramaniam & Woods 2016)
Serious Games (Wii)	(Portela et al. 2011)
Serious Games (Xbox Kinect)	(Chen et al. 2012)
**1.7. Studies on Process Planning/Work Process Management**	
Software for planning and optimizing nursing processes	(Pare et al. 2011)
Intelligent performance assessment system	(Valerie et al. 2016)
**1.8. Studies on Target Group Specific Interfaces**	
Electronic Medical Record Interface for ICU-use	(Olchanski et al. 2017)
Interface for people with memory impairment/dementia	(Lazar et al. 2016)
Dashboard design within an electronic health record	(Schall et al. 2017)
**2. Studies on Robotic Technologies**	
Physical assistance (robotic lifting device)	(Ranasinghe et al. 2014)
Physical assistance (robotic wheelchair)	(Wang et al. 2011)
Physical assistance/ Transport (pharmacy delivery robot)	(Summerfield et al. 2011)
Social/service robot (Cafero)	(Broadbent et al. 2015)
Social/telepresence robot	(Bettinelli et al. 2015)
Socially interactive robot (Guide robot, Cafero)	(Broadbent et al. 2016)
Social/therapeutic robot (JustoCat)	(Gustafsson et al. 2015)
Social/therapeutic robot (Paro, Pleo)	(Baisch et al. 2018)
Social/therapeutic robot (Paro)	(Moyle et al. 2017), (Petersen et al. 2017), (Robinson et al. 2013), (Jøranson et al. 2015), (Jøranson et al. 2016), (Bemelmans et al. 2015), (Liang et al. 2017), (Moyle et al. 2013), (Bennett et al. 2015) (Birks et al. 2016), (Šabanović et al. 2013), (Wagemaker et al. 2017), (Iacono & Marti 2016), (Wada et al. 2014)
Social/therapeutic robot (Paro) / humanoid socially assistive robot (NAO)	(Valenti et al. 2015), (Shukla et al. 2017)
**3. Studies on Sensors / Monitoring**	
Behaviour Analysis / Emergency detection<br>Behaviour Analysis / fall prevention	(van der Lende et al. 2016), (Hardin et al. 2013), (Sahota et al. 2014), (Shee et al. 2014), (Tchalla et al. 2013)
Behaviour Analysis / pressure ulcer prevention	(Pickham et al. 2018)
Behaviour Analysis of Carers/Hand hygiene	(Marra et al. 2014)
External risk detection /noise sensor	(Jousselme et al. 2011)
General Behaviour Analysis/ Decision support	(Lexis 2013), (Rantz et al. 2017), (Lazarou et al. 2016)
Tracking /GPS-Device	(Pot et al. 2012)
Tracking/RFID-Identification	(Osaimi et al. 2017)
Vital sign monitoring (patient)	(Brown et al. 2014), (Zhou et al. 2012), (Kuroda et al. 2013), (Pigini et al. 2017)
**4. Studies on Assistive Devices**	
Care support (multi-modal distraction)	(Miller et al. 2011)
Care support with treatment focus (smart pumps)	(Orto et al. 2015)<br>(Vadiei et al. 2017)
Care support for Activities of Daily Living (Drink monitoring)	(Zimmermann et al. 2017)
Reminder System (medication dispenser)	(Marek et al. 2013), (Akiyama & Sasaki 2013), (Suzuki et al. 2011)
**5. Studies on Ambient Assisted Living Solutions**	
AAL at home	(Hattink et al. 2016), (Nijhof et al. 2013b)
AAL at home incl. formal care	(Trukeschitz B. 2018)

6. Studies on Virtual Reality

Virtual Reality for distraction/pain reduction	(Kipping et al. 2012), (Mazzacano et al. 2016), (Patterson et al. 2012)

References

Akiyama, M. & Sasaki, Y. (2013). Efficacy of the drug administration support system for improving drug compliance in home-care. 2013 Proceedings of PICMET '13: Technology Management in the IT-Driven Services (PICMET).

Alexander, G.L., Pasupathy, K.S., Steege, L.M., Strecker, E.B. & Carley, K.M. (2014). Multi-disciplinary communication networks for skin risk assessment in nursing homes with high IT sophistication. International Journal of Medical Informatics, 83(8), 581-591.

Alexander, G.L., Steege, L.M., Pasupathy, K.S. & Wise, K. (2015). Case studies of IT sophistication in nursing homes: A mixed method approach to examine communication strategies about pressure ulcer prevention practices. International Journal of Industrial Ergonomics, 49, 156-166.

Angst, C.M., Devaraj, S. & D'Arcy, J. (2012). Dual role of IT-assisted communication in patient care: A validated structure-process-outcome framework. Journal of Management Information Systems, 29(2), 257-292.

Appari, A., Carian, E.K., Johnson, M.E. & Anthony, D.L. (2012). Medication administration quality and health information technology: a national study of US hospitals. Journal of the American Medical Informatics Association, 19(3), 360-367.

Appari, A., Johnson, E.M. & Anthony, D.L. (2014). Information technology and hospital patient safety: a cross-sectional study of US acute care hospitals. The American journal of managed care, 20(17), eSP39-eSP47.

Baisch, S., Kolling, T., Rühl, S., Klein, B., Pantel, J., Oswald, F. & Knopf, M. (2018). Emotional robots in a nursing context: Empirical analysis of the present use and the effects of Paro and Pleo. Zeitschrift für Gerontologie und Geriatrie, 51(1), 16-24.

Bemelmans, R., Gelderblom, G.J., Jonker, P. & de Witte, L. (2015). Effectiveness of Robot Paro in Intramural Psychogeriatric Care: A Multicenter Quasi-Experimental Study. Journal of the American Medical Directors Association, 16(11), 946-950.

Bennett, K., Grasso, F., Lowers, V., McKay, A. & Milligan, C. (2015). Evaluation of an App to Support Older Adults with Wounds. Proceedings of the 5th International Conference on Digital Health 2015, New York, NY, USA, ACM.

Berenbaum, R., Lange, Y. & Abramowitz, L. (2011). Augmentative Alternative Communication for Alzheimer?s Patients and Families? Using SAVION. Proceedings of the 4th International Conference on PErvasive Technologies Related to Assistive Environments, New York, NY, USA, ACM.

Bettinelli, M., Lei, Y., Beane, M., Mackey, C. & Liesching, T. (2015) Does Robotic Telerounding Enhance Nurse-Physician Collaboration Satisfaction About Care Decisions? Telemedicine journal and e-health 21, 637-643 10.1089/tmj.2014.0162: 10.1089/tmj.2014.0162.

Birks, M., Bodak, M., Barlas, J., Harwood, J. & Pether, M. (2016). Robotic Seals as Therapeutic Tools in an Aged Care Facility: A Qualitative Study. Journal of Aging Research, 2016.

Blakey, J.D., Guy, D., Simpson, C., Fearn, A., Cannaby, S., Wilson, P. & Shaw, D. (2012). Multimodal observational assessment of quality and productivity benefits from the implementation of wireless technology for out of hours working. BMJ Open, 2(2).

Bowles, K.H., Hanlon, A.L., Glick, H.A., Naylor, M.D., O'Connor, M., Riegel, B., Shih, N.W. & Weiner, M.G. (2011). Clinical effectiveness, access to, and satisfaction with care using a telehomecare substitution intervention: a randomized controlled trial. Int J Telemed Appl, 2011, 540138.

Bradley, S.L. (2011). A phenomenological exploration of nurses' perceptions of the effect of electronic documentation on healing relationships: University of Phoenix.

Broadbent, E., Kerse, N., Peri, K., Robinson, H., Jayawardena, C., Kuo, T., Datta, C., Stafford, R., Butler, H., Jawalkar, P., Amor, M., Robins, B. & MacDonald, B. (2016). Benefits and problems of health-care robots in aged care settings: A comparison trial. Australas J Ageing, 35(1), 23-29.

Broadbent, E., Orejana, J.R., Ahn, H.S., Xie, J., Rouse, P. & MacDonald, B.A. (2015). The cost-effectiveness of a robot measuring vital signs in a rural medical practice. 2015 24th IEEE International Symposium on Robot and Human Interactive Communication (RO-MAN).

Brown, H., Terrence, J., Vasquez, P., Bates, D.W. & Zimlichman, E. (2014). Continuous monitoring in an inpatient medical-surgical unit: a controlled clinical trial. The American journal of medicine, 127(3), 226-232.

Cady, R.G. (2012). Measuring the Impact of Technology on Nurse Workflow: A Mixed Methods Approach: University of Minnesota.

Cady, R.G. & Finkelstein, S.M. (2014). Task-technology fit of video telehealth for nurses in an outpatient clinic setting. Telemedicine journal and e-health : the official journal of the American Telemedicine Association, 20(7), 633-639.

Chanyagorn, P., Kungwannarongkun, B. & Chanyagorn, W. (2016). Design of electronic nursing Kardex system for medication error prevention in IPD patients. 2016 6th IEEE International Conference on Control System, Computing and Engineering (ICCSCE).

Chen, S.T., Huang, Y.G.L. & Chiang, I.T. (2012). Using Somatosensory Video Games to Promote Quality of Life for the Elderly with Disabilities. 2012 IEEE Fourth International Conference On Digital Game And Intelligent Toy Enhanced Learning.

Chiang, K.F. & Wang, H.H. (2016). Nurses' experiences of using a smart mobile device application to assist home care for patients with chronic disease: A qualitative study. Journal of Clinical Nursing, 25(13-14), 2008-2017.

Ching, J.M., Williams, B.L., Idemoto, L.M. & Blackmore, C.C. (2014). Using Lean 'Automation with a Human Touch' to Improve Medication Safety: A Step Closer to the 'Perfect Dose'. Joint Commission Journal on Quality & Patient Safety, 40(8), 341-350.

Chuang, S.T., Liu, Y.F., Fu, Z.X., Liu, K.C., Chien, S.H., Lin, C.L. & Lin, P.Y. (2015). Application of a smartphone nurse call system for nursing care. Telemedicine journal and e-health : the official journal of the American Telemedicine Association, 21(2), 105-109.

Clarke, C.N., Patel, S.H., Day, R.W., George, S., Sweeney, C., Monetes De Oca, G.A., Aiss, M.A., Grubbs, E.G., Bednarski, B.K., Lee, J.E., Bodurka, D.C., Skibber, J.M. & Aloia, T.A. (2017). Implementation of a standardized electronic tool improves compliance, accuracy, and efficiency of trainee-to-trainee patient care handoffs after complex general surgical oncology procedures. Surgery (United States), 161(3), 869-875.

Dykes, P.C., I-Ching, E.H., Soukup, J.R., Chang, F. & Lipsitz, S. (2012). A case control study to improve accuracy of an electronic fall prevention toolkit. AMIA ... Annual Symposium proceedings / AMIA Symposium. AMIA Symposium, 2012, 170-179.

Florczak, B., Scheurich, A., Croghan, J., Sheridan Jr, P., Kurtz, D., McGill, W. & McClain, B. (2012). An observational study to assess an electronic point-of-care wound documentation and reporting system regarding user satisfaction and potential for improved care. Ostomy Wound Management, 58(3), 46-51.

Göransson, C., Eriksson, I., Ziegert, K., Wengström, Y., Langius-Eklöf, A., Brovall, M., Kihlgren, A. & Blomberg, K. (2017). Testing an app for reporting health concerns-Experiences from older people and home care nurses. International Journal of Older People Nursing.

Gustafsson, C., Svanberg, C. & Müllersdorf, M. (2015). Using a Robotic Cat in Dementia Care. Journal of Gerontological Nursing, 41(10), 46-56.

Hardin, Sr., Dienemann, J., Rudisill, P. & Mills, K. (2013) Inpatient fall prevention: use of in-room Webcams. Journal of patient safety 9, 29-35 10.1097/PTS.0b013e3182753e4f: 10.1097/PTS.0b013e3182753e4f.

Hattink, B.J.J., Meiland, F.J.M., Overmars-Marx, T., de Boer, M., Ebben, P.W.G., van Blanken, M., Verhaeghe, S., Stalpers-Croeze, I., Jedlitschka, A., Flick, S.E., v/d Leeuw, J., Karkowski, I. & Dröes, R.M. (2016). The electronic, personalizable Rosetta system for dementia care: exploring the user-friendliness, usefulness and impact. Disability & Rehabilitation: Assistive Technology, 11(1), 61-71.

Hicken, B.L., Daniel, C., Luptak, M., Grant, M., Kilian, S. & Rupper, R.W. (2017). Supporting Caregivers of Rural Veterans Electronically (SCORE). Journal of Rural Health, 33(3), 305-313.

Hitt, L.M. & Tambe, P. (2016). Health care information technology, work organization, and nursing home performance. Industrial and Labor Relations Review, 69(4), 834-859.

Huang, H.-Y. & Lee, T.-T. (2011). Impact of bar-code medication administration on nursing activity patterns and usage experience in Taiwan. CIN: Computers, Informatics, Nursing, 29(10), 554-563.

Iacono, I. & Marti, P. (2016). Narratives and emotions in seniors affected by dementia: A comparative study using a robot and a toy. 2016 25th IEEE International Symposium on Robot and Human Interactive Communication (RO-MAN).

Jøranson, N., Pedersen, I., Rokstad, A.M.M. & Ihlebæk, C. (2015). Effects on Symptoms of Agitation and Depression in Persons With Dementia Participating in Robot-Assisted Activity: A Cluster-Randomized Controlled Trial. Journal of the American Medical Directors Association, 16(10), 867-873.

Jøranson, N., Pedersen, I., Rokstad, A.M.M. & Ihlebæk, C. (2016). Change in quality of life in older people with dementia participating in Paro-activity: a cluster-randomized controlled trial. Journal of Advanced Nursing, 72(12), 3020-3033.

Jousselme, C., Vialet, R., Jouve, E., Lagier, P., Martin, C. & Michel, F. (2011) Efficacy and mode of action of a noise-sensor light alarm to decrease noise in the pediatric intensive care unit: a prospective, randomized study. Pediatric critical care medicine 12, e69-72 10.1097/PCC.0b013e3181e89d91: 10.1097/PCC.0b013e3181e89d91.

Kipping, B., Rodger, S., Miller, K. & Kimble, R.M. (2012). Virtual reality for acute pain reduction in adolescents undergoing burn wound care: a prospective randomized controlled trial. Burns (03054179), 38(5), 650-657.

Kuroda, T., Noma, H., Naito, C., Tada, M., Yamanaka, H., Takemura, T., Nin, K. & Yoshihara, H. (2013). Prototyping sensor network system for automatic vital signs collection: Evaluation of a location based automated assignment of measured vital signs to patients. Methods of Information in Medicine, 52(3), 239-249.

Lang, R.L.N. (2012). Evaluating the Effectiveness of Nurse-Focused Computerized Clinical Decision Support on Urinary Catheter Practice Guidelines: Gardner-Webb University.

Lapane, K.L., Hughes, C.M., Daiello, L.A., Cameron, K.A. & Feinberg, J. (2011). Effect of a Pharmacist-Led Multicomponent Intervention Focusing on the Medication Monitoring Phase to Prevent Potential Adverse Drug Events in Nursing Homes. Journal of the American Geriatrics Society, 59(7), 1238-1245.

Lazar, A., Demiris, G. & Thompson, H.J. (2016). Evaluation of a multifunctional technology system in a memory care unit: Opportunities for innovation in dementia care. Informatics for Health & Social Care, 41(4), 373-386.

Lazarou, I., Karakostas, A., Stavropoulos, T.G., Tsompanidis, T., Meditskos, G., Kompatsiaris, I. & Tsolaki, M. (2016). A Novel and Intelligent Home Monitoring System for Care Support of Elders with Cognitive Impairment. Journal of Alzheimer's Disease, 54(4), 1561-1591.

Lear, C.L. & Walters, C. (2015). Use of Electronic Nurse Reminders to Improve Documentation. CIN: Computers, Informatics, Nursing, 33(12), 523-529.

Lexis, M. (2013). Activity monitoring technology to support homecare delivery to frail and psychogeriatric elderly persons living at home alone. Technology & Disability, 25(3), 189-197.

Liang, A., Piroth, I., Robinson, H., MacDonald, B., Fisher, M., Nater, U.M., Skoluda, N. & Broadbent, E. (2017). A Pilot Randomized Trial of a Companion Robot for People With Dementia Living in the Community. Journal of the American Medical Directors Association, 18(10), 871-878.

Lo, Y.S., Lee, W.S., Chen, G.B. & Liu, C.T. (2014). Improving the work efficiency of healthcare-associated infection surveillance using electronic medical records. Computer Methods and Programs in Biomedicine, 117(2), 351-359.

Marek, K., Stetzer, F., Ryan, P., Bub, L., Adams, S., Schlidt, A., Lancaster, R. & O'Brien, A. (2013) Nurse care coordination and technology effects on health status of frail older adults via enhanced self-management of medication: randomized clinical trial to test efficacy. Nursing research 62, 269-278 10.1097/NNR.0b013e318298aa55: 10.1097/NNR.0b013e318298aa55.

Marra, A.R., Sampaio Camargo, T.Z., Magnus, T.P., Blaya, R.P., dos Santos, G.B., Guastelli, L.R., Rodrigues, R.D., Prado, M., Victor, E.d.S., Bogossian, H., Monte, J.C.M., dos Santos, O.F.P.o., Oyama, C.K. & Edmond, M.B. (2014). The use of real-time feedback via wireless technology to improve hand hygiene compliance. American Journal of Infection Control, 42(6), 608-611.

Mazzacano, S.D., McSherry, T., Atterbury, M., Helmold, E., Gartner, S. & Schulman, C. (2016) Effect of virtual reality distraction therapy on pain and anxiety in adult patients undergoing complex dressing changes: a randomized controlled trial. Journal of burn care and research. 37, S157

McKenna, R.M., Dwyer, D. & Rizzo, J.A. (2017). Is HIT a hit? The impact of health information technology on inpatient hospital outcomes. Applied Economics, 1-13.

Meehan, R. (2017). Electronic Health Records in Long-Term Care: Staff Perspectives. Journal of Applied Gerontology, 36(10), 1175-1196.

Melby, L., Brattheim, B.J. & Hellesø, R. (2015). Patients in transition - improving hospital-home care collaboration through electronic messaging: providers' perspectives. Journal of Clinical Nursing, 24(23/24), 3389-3399.

Meyer-Delpho, C. & Schubert, H.J. (2014). Potential of Information and Communications Technology to Improve Intersectoral Processes of Care: A Case Study of the Specialised Outpatient Palliative Care. Gesundheitswesen, 77(8-9), 550-556.

Mierlo, L., Meiland, F., Ven, P., Hout, H. & Dröes, R. (2015) Evaluation of DEM-DISC, customized e-advice on health and social support services for informal carers and case managers of people with dementia; a cluster randomized trial. International Psychogeriatrics 27, 1365-1378 10.1017/S1041610215000423: 10.1017/S1041610215000423.

Miller, K., Rodger, S., Kipping, B. & Kimble, R.M. (2011). A novel technology approach to pain management in children with burns: A prospective randomized controlled trial. Burns (03054179), 37(3), 395-405.

Mitchell, S. & Yaylacicegi, U. (2012). EHR prescription for small, medium, and large hospitals: an exploratory study of Texas acute care hospitals. Int J Electron Healthc, 7(2), 125-140.

Moyle, W., Cooke, M., Beattie, E., Jones, C., Klein, B., Cook, G. & Gray, C. (2013) Exploring the effect of companion robots on emotional expression in older adults with dementia: a pilot randomized controlled trial. Journal of Gerontological Nursing 39, 46-53 10.3928/00989134-20130313-03: 10.3928/00989134-20130313-03.

Moyle, W., Jones, C.J., Murfield, J.E., Thalib, L., Beattie, E.R.A., Shum, D.K.H., O'Dwyer, S.T., Mervin, M.C. & Draper, B.M. (2017). Use of a Robotic Seal as a Therapeutic Tool to Improve Dementia Symptoms: A Cluster-Randomized Controlled Trial. Journal of the American Medical Directors Association, 18(9), 766-773.

Munyisia, E.N., Yu, P. & Hailey, D. (2012). The impact of an electronic nursing documentation system on efficiency of documentation by caregivers in a residential aged care facility. Journal of Clinical Nursing, 21(19/20), 2940-2948.

Nijhof, N., van Gemert-Pijnen, J.E.W.C., Burns, C.M. & Seydel, E.R. (2013a). A personal assistant for dementia to stay at home safe at reduced cost. Gerontechnology, 11(3), 469-479.

Nijhof, N., van Gemert-Pijnen, L.J., Woolrych, R. & Sixsmith, A. (2013b). An evaluation of preventive sensor technology for dementia care. Journal of Telemedicine and Telecare, 19(2), 95-100.

Nordheim, J., Hamm, S., Kuhlmey, A. & Suhr, R. (2015). Tablet computers and their benefits for nursing home residents with dementia: Results of a qualitative pilot study. Zeitschrift für Gerontologie und Geriatrie, 48(6), 543-549.

Oakley, B. & Hunter, J.B. (2017). Implementing an electronic patient handover system. British Journal of Hospital Medicine (17508460), 78(1), 16-19.

Olchanski, N., Dziadzko, M.A., Tiong, I.C., Daniels, C.E., Peters, S.G., O'Horo, J.C. & Gong, M.N. (2017). Can a Novel ICU Data Display Positively Affect Patient Outcomes and Save Lives? Journal of Medical Systems, 41(11).

Orto, V., Hendrix, C.C., Griffith, B. & Shaikewitz, S.T. (2015). Implementation of a Smart Pump Champions Program to Decrease Potential Patient Harm. Journal of Nursing Care Quality, 30(2), 138-143.

Osaimi, A.A.A., Kadi, K.A. & Saddik, B. (2017). Role of radio frequency identification in improving infant safety and the extent of nursing staff acceptance of RFID at King Abdulaziz medical city in Riyadh. 2017 International Conference on Informatics, Health & Technology (ICIHT).

Paranilam, S.O. (2013). Effectiveness of an Electronic Pain Notification System on Postoperative Pain: University of Maryland, Baltimore.

Paré, G., Poba-Nzaou, P. & Sicotte, C. (2013). Home telemonitoring for chronic disease management: An economic assessment. International Journal of Technology Assessment in Health Care, 29(2), 155-161.

Pare, G., Sicotte, C., Moreault, M.P., Poba-Nzaou, P., Templier, M. & Nahas, G. (2011). Effects of Mobile Computing on the Quality of Homecare Nursing Practice. 2011 44th Hawaii International Conference on System Sciences.

Patmon, F.L., Gee, P.M., Rylee, T.L. & Readdy, N.L. (2016). Using Interactive Patient Engagement Technology in Clinical Practice: A Qualitative Assessment of Nurses' Perceptions. J Med Internet Res, 18(11), e298.

Patterson, D., Soltani, M., Teeley, A., Morse, D., Wiechman, S. & Gibran, N. (2012) Hypnosis delivered through immersive virtual reality for wound care: a randomized, controlled study. Journal of burn care and research. 33, S70

Pemmassani, V., Paget, T., van Woerden, H.C. & Pemmasani, S. (2014). Hands-free communication to free up nursing time. Nursing Times, 110(13), 12-14.

Petersen, S., Houston, S., Qin, H., Tague, C. & Studley, J. (2017) The Utilization of Robotic Pets in Dementia Care. Journal of Alzheimer's Disease 55, 569-574 10.3233/JAD-160703: 10.3233/JAD-160703.

Pickham, D., Berte, N., Pihulic, M., Valdez, A., Mayer, B. & Desai, M. (2018). Effect of a wearable patient sensor on care delivery for preventing pressure injuries in acutely ill adults: A pragmatic randomized clinical trial (LS-HAPI study). International journal of nursing studies, 80, 12-19.

Pigini, L., Bovi, G., Panzarino, C., Gower, V., Ferratini, M., Andreoni, G., Sassi, R., Rivolta, M.W. & Ferrarin, M. (2017). Pilot Test of a New Personal Health System Integrating Environmental and Wearable Sensors for Telemonitoring and Care of Elderly People at Home (SMARTA Project). Gerontology, 63(3), 281-286.

Portela, F.R., Correia, R.J.C., Fonseca, J.A. & Andrade, J.M. (2011). Wiitherapy on seniors - Effects on physical and metal domains. 2011 IEEE 1st International Conference on Serious Games and Applications for Health (SeGAH).

Pot, A.M., Willemse, B.M. & Horjus, S. (2012). A pilot study on the use of tracking technology: Feasibility, acceptability, and benefits for people in early stages of dementia and their informal caregivers. Aging & Mental Health, 16(1), 127-134.

Ranasinghe, R., Dantanarayana, L., Tran, A., Lie, S., Behrens, M. & Liu, L. (2014). Smart hoist: An assistive robot to aid carers. 2014 13th International Conference on Control Automation Robotics & Vision (ICARCV).

Rantz, M., Phillips, L.J., Galambos, C., Lane, K., Alexander, G.L., Despins, L., Koopman, R.J., Skubic, M., Hicks, L., Miller, S., Craver, A., Harris, B.H. & Deroche, C.B. (2017). Randomized Trial of Intelligent Sensor System for Early Illness Alerts in Senior Housing. Journal of the American Medical Directors Association, 18(10), 860-870.

Rantz, M.J., Alexander, G., Galambos, C., Flesner, M.K., Vogelsmeier, A., Hicks, L., Scott-Cawiezell, J., Zwygart-Stauffacher, M. & Greenwald, L. (2011). The use of bedside electronic medical record to improve quality of care in nursing facilities: a qualitative analysis. CIN: Computers, Informatics, Nursing, 29(3), 149-156.

Restuccia, J.D., Cohen, A.B., Horwitt, J.N. & Shwartz, M. (2012). Hospital implementation of health information technology and quality of care: Are they related? BMC Medical Informatics and Decision Making, 12(1).

Robinson, H., MacDonald, B., Kerse, N. & Broadbent, E. (2013). The Psychosocial Effects of a Companion Robot: A Randomized Controlled Trial. Journal of the American Medical Directors Association, 14(9), 661-667.

Rodriguez, C.S. (2016). ENHANCING THE COMMUNICATION OF SUDDENLY SPEECHLESS CRITICAL CARE PATIENTS. American Journal of Critical Care, 25(3), e40-e47.

Šabanović, S., Bennett, C.C., Chang, W.L. & Huber, L. (2013). PARO robot affects diverse interaction modalities in group sensory therapy for older adults with dementia. 2013 IEEE 13th International Conference on Rehabilitation Robotics (ICORR).

Sahota, O., Drummond, A., Kendrick, D., Grainge, M.J., Vass, C., Sach, T., Gladman, J. & Avis, M. (2014). REFINE (REducing Falls in In-patieNt Elderly) using bed and bedside chair pressure sensors linked to radio-pagers in acute hospital care: a randomised controlled trial. Age Ageing, 43(2), 247-253.

Salinas, J., Chung, K.K., Mann, E.A., Cancio, L.C., Kramer, G.C., Serio-Melvin, M.L., Renz, E.M., Wade, C.E. & Wolf, S.E. (2011). Computerized decision support system improves fluid resuscitation following severe burns: An original study. Critical Care Medicine, 39(9), 2031-2038.

Schall, M.C., Cullen, L., Pennathur, P., Chen, H., Burrell, K. & Matthews, G. (2017). Usability Evaluation and Implementation of a Health Information Technology Dashboard of Evidence-Based Quality Indicators. CIN: Computers, Informatics, Nursing, 35(6), 281-287.

Seibert, H.H., Maddox, R.R., Flynn, E.A. & Williams, C.K. (2014). Effect of barcode technology with electronic medication administration record on medication accuracy rates. American Journal of Health-System Pharmacy, 71(3), 209-218.

Shee, A.W., Phillips, B., Hill, K. & Dodd, K. (2014). Feasibility, acceptability, and effectiveness of an electronic sensor bed/chair alarm in reducing falls in patients with cognitive impairment in a subacute ward. Journal of Nursing Care Quality, 29(3), 253-262.

Shukla, J., Barreda-Ángeles, M., Oliver, J. & Puig, D. (2017). Effectiveness of socially assistive robotics during cognitive stimulation interventions: Impact on caregivers. 2017 26th IEEE International Symposium on Robot and Human Interactive Communication (RO-MAN).

Steurbaut, K., Colpaert, K., Van Hoecke, S., Steurbaut, S., Danneels, C., Decruyenaere, J. & De Turck, F. (2012). Design and evaluation of a service oriented architecture for paperless ICU tarification. Journal of Medical Systems, 36(3), 1403-1416.

Steventon, A., Bardsley, M., Billings, J., Dixon, J., Doll, H., Beynon, M., Hirani, S., Cartwright, M., Rixon, L., Knapp, M., Henderson, C., Rogers, A., Hendy, J., Fitzpatrick, R. & Newman, S. (2013). Effect of telecare on use of health and social care services: findings from the Whole Systems Demonstrator cluster randomised trial. Age Ageing, 42(4), 501-508.

Subramaniam, P. & Woods, B. (2016) Digital life storybooks for people with dementia living in care homes: an evaluation. Clinical interventions in aging 11, 1263-1276 10.2147/CIA.S111097: 10.2147/CIA.S111097.

Summerfield, M.R., Seagull, F.J., Vaidya, N. & Xiao, Y. (2011). Use of pharmacy delivery robots in intensive care units. American Journal of Health-System Pharmacy, 68(1), 77-83.

Suzuki, S., Yokoishi, T., Hada, H., Mitsugi, J., Nakamura, O. & Murai, J. (2011). Bidirectional medication support system for medical staff and home care patients. 2011 5th International Symposium on Medical Information and Communication Technology.

Takian, A., Sheikh, A. & Barber, N. (2012). We are bitter, but we are better off: Case study of the implementation of an electronic health record system into a mental health hospital in England. BMC Health Services Research, 12(1).

Tchalla, A.E., Lachal, F., Cardinaud, N., Saulnier, I., Rialle, V. & Preux, P.-M. (2013) Preventing and managing indoor falls with home-based technologies in mild and moderate Alzheimer's disease patients: pilot study in a community dwelling. Dementia and geriatric cognitive disorders 36, 251-261 10.1159/000351863: 10.1159/000351863.

Tielbur, B.R., Rice Cella, D.E., Currie, A., Roach, J.D., Mattingly, B., Boone, J., Watwood, C., McGauran, A., Kirshner, H.S. & Charles, P.D. (2015). Discharge huddle outfitted with mobile technology improves efficiency of transitioning stroke patients into follow-up care. American Journal of Medical Quality, 30(1), 36-44.

Trukeschitz B., S.C., Ring-Dimitriou S. (2018). Smartes Betreutes Wohnen: Nutzung, Systemakzeptanz und Wirkungen von „meinZentrAAL“. Norderstedt, Deutschland.

Vadiei, N., Shuman, C., Murthy, M. & Daley, M. (2017) Optimization of intelligent infusion pump technology to minimize vasopressor pump programming errors. Expert opinion on drug safety, 1-5 10.1080/14740338.2017.1323866: 10.1080/14740338.2017.1323866.

Valenti, S.M., Aguera-Ortiz, L., Olazaran, R.J., Mendoza, R.C., Perez, M.A., Rodriguez, P.I., Osa, R.E., Barrios, S.A., Herrero, C.V., Carrasco, C.L., Felipe, R.S., Lopez, A.J., Leon, S.B., Canas, P.J., Martin, R.F. & Martinez, M.P. (2015) Social robots in advanced dementia. Frontiers in Aging Neuroscience 7, 10.3389/fnagi.2015.00133: 10.3389/fnagi.2015.00133.

Valerie, T., Choy, K.L., Siu, P.K.Y., Lam, H.Y., Ho, G.T.S. & Cheng, S.W.Y. (2016). An intelligent performance assessment system for enhancing the service quality of home care nursing staff in the healthcare industry. 2016 Portland International Conference on Management of Engineering and Technology (PICMET).

van der Heide, L.A., Willems, C.G., Spreeuwenberg, M.D., Rietman, J. & de Witte, L.P. (2012). Implementation of CareTV in care for the elderly: The effects on feelings of loneliness and safety and future challenges. Technology & Disability, 24(4), 283-291.

van der Lende, M., Cox, F.M.E., Visser, G.H., Sander, J.W. & Thijs, R.D. (2016). Value of video monitoring for nocturnal seizure detection in a residential setting. Epilepsia, 57(11), 1748-1753.

Vowden, K. & Vowden, P. (2013). A pilot study on the potential of remote support to enhance wound care for nursing-home patients. J Wound Care, 22(9), 481-488.

Wada, K., Takasawa, Y. & Shibata, T. (2014). Robot therapy at facilities for the elderly in Kanagawa prefecture - a report on the experimental result of the first month. The 23rd IEEE International Symposium on Robot and Human Interactive Communication.

Wagemaker, E., Dekkers, T.J., Agelink van Rentergem, J.A., Volkers, K.M. & Huizenga, H.M. (2017). Advances in Mental Health Care: Five N = 1 Studies on the Effects of the Robot Seal Paro in Adults With Severe Intellectual Disabilities. Journal of Mental Health Research in Intellectual Disabilities, 10(4), 309-320.

Wakefield, B.J. & Vaughan-Sarrazin, M. (2017). Home Telehealth and Caregiving Appraisal in Chronic Illness. Telemedicine and e-Health, 23(4), 282-289.

Wang, R.H., Gorski, S.M., Holliday, P.J. & Fernie, G.R. (2011). Evaluation of a Contact Sensor Skirt for an Anti-Collision Power Wheelchair for Older Adult Nursing Home Residents With Dementia: Safety and Mobility. Assistive technology, 23(3), 117-134.

Webster, G. & Hanson, V.L. (2014). Technology for Supporting Care Staff in Residential Homes. ACM Trans. Access. Comput., 5(3), 8-1.

White, C., McIlfatrick, S., Dunwoody, L. & Watson, M. (2015). Supporting and improving community health services-a prospective evaluation of ECHO technology in community palliative care nursing teams. BMJ Support Palliat Care.

Wieck, M., Blake, B., Sellick, C., Kenron, D., DeVries, D., Terry, S. & Krishnaswami, S. (2017) Utilizing technology to improve intraoperative family communication. American journal of surgery. (no pagination), 2017 Date of Publication: January 10, 10.1016/j.amjsurg.2017.03.014: 10.1016/j.amjsurg.2017.03.014.

Wu, R., Rossos, P., Quan, S., Reeves, S., Lo, V., Wong, B., Cheung, M. & Morra, D. (2011). An evaluation of the use of smartphones to communicate between clinicians: A mixed-methods study. Journal of Medical Internet Research, 13(3).

Yeaman, B., Ko, K.J. & Castillo, R.A.d. (2015). Care Transitions in Long-term Care and Acute Care: Health Information Exchange and Readmission Rates. Online Journal of Issues in Nursing, 20(3), 1-1.

Yi-Sheng, C., Hsin-Ju, L. & Yuan-Hsiang, L. (2014). Using wireless measuring devices and Tablet PC to improve the efficiency of vital signs data collection in hospital. 2014 IEEE International Symposium on Bioelectronics and Bioinformatics (IEEE ISBB 2014).

Yusof, M.M. (2015). A case study evaluation of a Critical Care Information System adoption using the socio-technical and fit approach. International Journal of Medical Informatics, 84(7), 486-499.

Zaccarelli, C., Cirillo, G., Passuti, S., Annicchiarico, R. & Barban, F. (2013). Computer-based cognitive intervention for dementia Sociable: motivating platform for elderly networking, mental reinforcement and social interaction. 2013 7th International Conference on Pervasive Computing Technologies for Healthcare and Workshops.

Zhou, J., Liu, D.B., Zhong, J.W., Huang, Z.Y., Qiu, S.Y., Zhou, Y.P. & Yi, X.H. (2012). Feasibility of a remote monitoring system for home-based non-invasive positive pressure ventilation of children and infants. International Journal of Pediatric Otorhinolaryngology, 76(12), 1737-1740.

Zhuang, J., Fang, R., Feng, X., Xu, X., Liu, L., Bai, Q., Tang, H., Zhao, Z. & Chen, S. (2013) The impact of human-computer interaction-based comprehensive training on the cognitive functions of cognitive impairment elderly individuals in a nursing home. Journal of Alzheimer's Disease 36, 245-251 10.3233/JAD-130158: 10.3233/JAD-130158.

Zimmermann, C., Zeilfelder, J., Bloecher, T., Diehl, M., Essig, S. & Stork, W. (2017). Evaluation of a smart drink monitoring device. 2017 IEEE Sensors Applications Symposium (SAS).
